# The Conundrum of Occult Cancer Screening in Venous Thromboembolism: Lessons from the REMOTEV Registry

**DOI:** 10.3390/medicina58070913

**Published:** 2022-07-09

**Authors:** Elena-Mihaela Cordeanu, Lucas Jambert, Jonathan Tousch, Corina Mirea, Alexandre Delatte, Waël Younes, Bastien Woehl, Claire Harter, Anne-Sophie Frantz, Amer Hamade, Valérie Schini-Kerth, Patrick Ohlmann, Emmanuel Andres, Dominique Stephan

**Affiliations:** 1 Department of Hypertension, Vascular Disease and Clinical Pharmacology, Strasbourg Regional University Hospital, 67091 Strasbourg, France; jambertlucas@gmail.com (L.J.); jonathan.tousch@orange.fr (J.T.); corina.mirea@chru-strasbourg.fr (C.M.); anne-sophie.frantz@chru-strasbourg.fr (A.-S.F.); dominique.stephan@chru-strasbourg.fr (D.S.); 2 Department of Cardiology, Haguenau Regional Hospital, 67500 Haguenau, France; delatte.alex@gmail.com; 3 Department of Vascular Medicine, Colmar Regional Hospital, 68000 Colmar, France; y_wael@hotmail.com; 4 Department of Vascular Medicine, Mulhouse Regional Hospital, 68100 Mulhouse, France; bastien.woehl@gmail.com (B.W.); hamadea@ghrmsa.fr (A.H.); 5 Department of Radiology, Strasbourg Regional University Hospital, 67091 Strasbourg, France; claire.harter@chru-strasbourg.fr; 6 UMR 1260 INSERM Regenerative Nanomedecine, Faculty of Pharmacy, Strasbourg University, 67400 Illkirch, France; valerie.schini-kerth@unistra.fr; 7 Cardiology Department, Strasbourg Regional University Hospital, 67091 Strasbourg, France; patrick.ohlmann@chru-strasbourg.fr; 8 Internal Medicine Department, Strasbourg Regional University Hospital, 67091 Strasbourg, France; emmanuel.andres@chru-strasbourg.fr

**Keywords:** occult cancer, venous thromboembolism, pulmonary embolism, screening

## Abstract

(1) *Background and Objectives*: Venous thromboembolism (VTE) is strongly associated with cancer, and may be the first event revealing occult neoplasia. Nonetheless, the reasonable extent of the etiological assessment after an unprovoked VTE event remains debated. The main objective of this study was to evaluate the incidence of occult neoplasia one year after an episode of VTE, in consecutively hospitalized patients for VTE from the REMOTEV registry. The secondary objectives were to assess the performance of the various tests used for occult cancer screening in a real-life setting and analyze the risk factors associated with the discovery of cancer and the 1-year prognosis. (2) *Methods*: REMOTEV is a prospective, non-interventional cohort study of patients with acute VTE. Patients included in the registry from 23 October 2013 to 28 July 2018 were analyzed after a follow-up of 12 months. Cancer detection was performed according to local practices and consisted of a limited strategy to which an abdominal ultrasound was added. In the presence of suggestive clinical manifestations, further examinations were performed on an individual basis. (3) *Results*: A total of 993 patients were included in the study. At 1 year, the incidence of newly diagnosed cancer was low (5.3%). Half of the detected cancers were metastatic at discovery (51%) and had a poor global prognosis (32% of mortality at 1 year). Admission pulmonary CT scans as well as (thoracic)-abdomino-pelvic CT scans (when performed) were responsible for the majority of detected cancers. Age over 65 years and the concomitant presence of an unusual site and lower-limb deep vein thrombosis were the only factors associated with occult neoplasia in this cohort. After 1-year FU, mortality was higher in cancer patients (HR 6.0 (CI 95% 3.5–10.3, *p* < 0.0001)), and cancer evolution was the leading cause of death in the cancer group. (4) *Conclusions*: In REMOTEV, VTE-revealed occult cancer prevalence was low, but similar to recent reports and associated with higher age, multiple thrombotic sites and worse prognosis.

## 1. Introduction

Active cancer is a recognized major risk factor for venous thromboembolism (VTE), with an odds ratio of 5–7 depending on the cancer type and extension [1]. VTE and cancer co-occurrence constitutes a two-way association: 25% of all VTE events are attributed to cancer, and up to 20% of cancer patients experience VTE at some stage of their evolution, with a higher incidence during the initial period after cancer diagnosis [2,3]. VTE pejoratively influences the course of cancer, with frequent recurrences despite therapeutic anticoagulation, thus being the second leading cause of death in cancer patients [4]. In a minority of VTE patients (5–10%), cancer is occult at the time of VTE diagnosis, which has led to numerous interrogations concerning the reasonable extent of cancer screening (limited versus extensive) following VTE, thus allowing earlier cancer detection and prognosis improvement. Limited screening currently refers to a thorough medical history and physical examination, standard laboratory tests (blood count, protein testing and urinalysis), chest X-ray and age- and sex-specific mass screening tests (mammogram, Pap test, fecal occult blood test (FOBD), prostatic-specific antigen (PSA) test). Extended screening includes, beyond the limited screening, morphological examinations such as ultrasound of the abdomen and pelvis, abdominal computed tomography (CT), endoscopies and sometimes whole-body positron emission tomography (PET/CT) [5]. Extensive strategies are less cost-effective and more prone to iatrogenic complications than limited ones. In 2004, Piccioli et al. showed that after a first unprovoked VTE event, followed by an extensive screening strategy including abdomino-pelvic CT scan and tumor markers systematically associated with endoscopic invasive tests (gastroscopy and colonoscopy), the frequency of detection of occult cancer was approximately 10% during a follow-up (FU) period of 2 years [6]. Other studies have shown that a limited screening, left to the choice of the physician, was associated with a 5% incidence of cancer [7]. Robin et al. did not show any significant benefit in performing a PET/CT versus a limited screening strategy (physical examination, usual laboratory tests and basic radiographs) in the diagnosis of occult malignancy following an unprovoked VTE [8]. In contrast, Carrier et al. showed that an extensive morphologic screening with an abdomino-pelvic CT scan allowed a significant increase in the diagnosis of occult cancer compared to a standard screening [9]. However, the SOME trial, comparing a limited screening to an extensive approach, including an abdominal CT associated with a virtual colonoscopy and gastroscopy, did not improve cancer detection or prognosis [10]. To date, although extensive screening procedures have been associated with a higher number of detected cancers, their impact on survival seems uncertain [11]. As such, all recent guidelines favor limited screening, but current real-life clinical practices are mainly based on individual screening strategies according to the physician’s choice [5]. As cancer-predictive scores appear misleading, the clinician’s decision remains empirically based on classical risk factors for cancer-associated thrombosis, such as bilateral deep vein thrombosis (DVT), unusual site thrombosis or recurrence during anticoagulation [12]. Faced with this quandary, we conducted a study of real-life cancer screening practices based on a prospective VTE registry.

## 2. Materials and Methods

### 2.1. Study Design and Patient Selection

REMOTEV is an ongoing, prospective registry enrolling all consecutive patients hospitalized in the Vascular Medicine Unit of Strasbourg University Hospital for acute DVT and/or pulmonary embolism (PE), with a one-year FU [13,14,15,16,17]. In the present study, we carried out an analysis of patients included between 23 October 2013 (starting date of the registry) and 28 July 2018 in order to evaluate the incidence of VTE-revealed cancers at 1 year after acute VTE. The secondary objectives were to assess the risk factors associated with the presence of occult cancer at the time of VTE diagnosis and the performance of additional etiological investigations. Patients were informed about the purpose of the registry and gave oral consent for participation, according to the requirements of the local Ethics Committee. Data were recorded in computerized, anonymized form. Patients with a history of cancer (cured or in remission) or a known active cancer at the time of VTE diagnosis were excluded.

### 2.2. Requirements for VTE Diagnosis

VTE diagnosis was established by a validated imaging test: CT pulmonary angiogram (CTPA) or ventilation–perfusion (VQ) lung scan for PE and venous compression ultrasound of the lower limbs for DVT. VTE was classified as provoked or unprovoked depending on the presence of major risk factors such as recent surgery, prolonged immobilization, pregnancy or a postpartum setting and estrogen therapy (oral contraception or hormone replacement therapy).

### 2.3. Baseline Variables

Age, sex, weight, height, cardiovascular risk factors, major comorbidities and common VTE risk factors were collected at baseline. Acute VTE characteristics (PE severity, DVT extension) and biological markers (Troponin, D-Dimers, BNP, creatinine, Cockcroft creatinine clearance, red blood cells count) were also colligated during initial hospitalization [13].

### 2.4. Occult Cancer Screening Modalities

Cancer detection was performed according to local practices (2014 ESC guidelines and 2017 ISTH guidelines) and consisted of a limited strategy to which an abdominal ultrasound was added in case of unprovoked events [5,18]. In the presence of suggestive clinical manifestations, further examinations were carried out on an individual basis. The following investigations were performed: chest X-ray (systematically performed in the emergency department), admission CTPA (for PE diagnosis), abdominal ultrasound (systematically performed in case of unprovoked VTE) and biological assessments, including a blood count (BC), serum protein electrophoresis (SPEP) and the search for occult gastrointestinal bleeding by performing a fecal occult blood test (FOBT). In addition, sex-dependent examinations were also carried out: a prostatic-specific antigen (PSA) test in men and a mammogram and Pap test in women. Secondary abdominal and pelvic CT scans, gastrointestinal endoscopy or PET/CT were performed according to first-line test results and clinical context. Cancer screening tests were carried out during hospitalization or on an outpatient basis, according to local practices. Diagnostic performance of cancer examinations was assessed through two ratios: (1) number of confirmed cancers/number of performed examinations and (2) number of confirmed cancers/number of positive tests.

### 2.5. Occult Cancer Diagnosis

Occult cancer diagnosis relied on biopsy confirmation. When malignancy histological proof was lacking (death before tissue biopsy, patients’ refusal for extensive testing), cancer diagnosis was accepted based on a set of clinical, biological and imaging features highly suggestive of cancer.

### 2.6. Follow-Up and Outcome Assessment

The observation period ended 12 months after the index event. All patient data were collected at the initial visit and then via phone interview at 1 month (±5 days), 3 months (±10 days), 6 months (±15 days) and 12 months (±15 days).

### 2.7. Statistical Analysis

This was a prospective observational study and therefore no formal power calculation was performed. Continuous variables were expressed as mean standard deviation (SD) or median with interquartile range (IQR) depending on the distribution. The normality of the distribution was assessed graphically and using the Shapiro–Wilk test. Categorical variables were presented as numbers of cases (percentages). Patients were divided according to the presence or absence of occult cancer. The association between occult cancer and baseline characteristics known to be correlated to cancer was assessed by univariate analysis. Risk factors associated with occult neoplasia having a univariate test considered significant (*p* < 0.10) were selected as a candidate for the multivariate logistic regression analysis. Results were expressed as odds ratio (ORs) with 95% confidence intervals (CI). We compared the risk of death and the risk of a composite criterion (all-cause death, major bleeding or VTE recurrence) between the two groups (“occult cancer” versus “cancer-free”). Results were expressed as hazard ratios (HRs) with 95% confidence intervals (CI). The Kaplan–Meier estimator was employed to compute survival curves over the 12-month follow-up (FU). A *p*-value < 0.05 was considered statistically significant. All analyses were performed using R software, version 3.2.2 (R project software: www.r-project.org: accessed on 25 March 2022). 

## 3. Results

### 3.1. Patient Characteristics at Baseline

**Figure 1:**
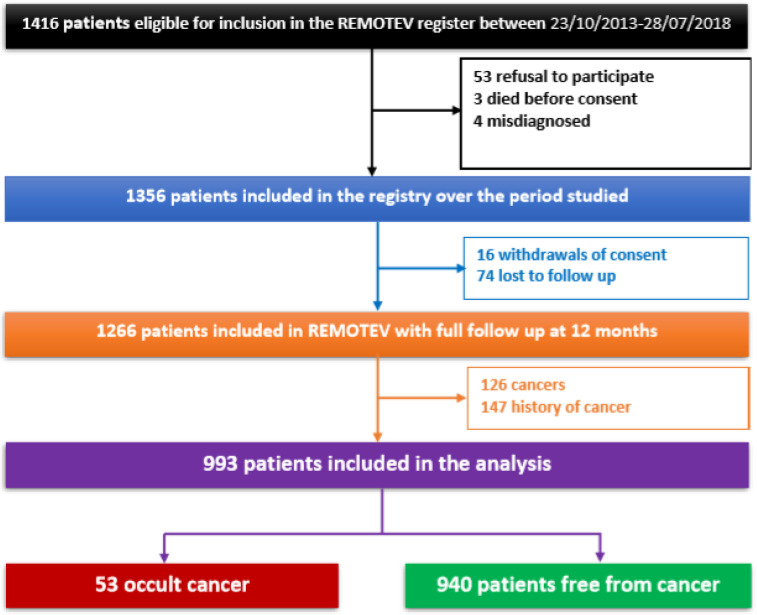
Study flowchart showing patients’ selection.

From October 2013 to July 2018, 1416 patients were eligible for inclusion in the REMOTEV registry. A total of 993 patients (53% females, mean age 63.5 ± 18.6 years old, 87% PE, 35% provoked VTE) were included in this analysis after the exclusion of patients who declined participation (n = 53), who died before being able to give their consent (n = 3), who were misdiagnosed (n = 4), who withdrew their consent during FU (n = 16) or were lost to FU (n = 74) and finally patients who had active cancer at baseline (n = 126) or a history of cancer (n = 147) (Figure 1). Among the 993 patients in our cohort, 53 (5.3%) were diagnosed with cancer within one year of inclusion in the registry. For the purpose of this study, the cohort was analyzed according to the discovery of previously unknown cancer following a VTE index event (“occult cancer” group: N = 53 vs. “occult cancer-free” group: N = 940). A comprehensive, comparative description of the baseline characteristics and index events of the two subgroups is presented in Table 1 and Table 2.

### 3.2. Etiological Assessment for Occult Cancer

A considerable proportion of previously unknown cancers were suspected on the initial CTPA. Cancer screening was then limited to sex- and age-specific cancer detection strategies but also depended on the clinical context, in which case the choice of further examination was left to the prescriber’s choice. Overall, at least one screening exam in search for underlying occult neoplasia was performed in 836 patients (84.1%) (Table 3).

The most frequently diagnosed cancers were cancers of the digestive tract (23%) and urinary tract (23%), and pulmonary (19%) and hematological neoplasia (19%). Twenty-seven (51%) were already metastatic at the time of discovery. Among the 53 cancers, 45 (85%) were diagnosed following an examination carried out as part of the initial etiological assessment. Among the other eight cancers, two were diagnosed during a bleeding complication (one glioblastoma responsible for neurological focal deficit during the first week following VTE, and one gastric cancer revealed by melena occurring 3 months after the index event), one pancreatic cancer was diagnosed after an episode of ascites infection, two skin cancers were diagnosed on the presence of suspicious skin lesions and, finally, three hematologic malignancies were diagnosed directly through the JAK2 mutation. After VTE diagnosis, the median time to suspect cancer was 2 days (IQR 1–6.25). For the 48 patients having pathology report confirmation, the median time for histological diagnosis was 29 days (IQR 8.5–95.5). The length of hospital stay was higher in the presence of occult cancer: 8 days (6–13) as compared to 6 days (4–8) in the cancer-free group (*p* < 0.0001).

One third of all cancers diagnosed in our study (30.2%) were suspected on the *initial CTPA* performed for VTE diagnosis (N = 627). Among these initial chest CT scans, 16 identified lesions that were further confirmed as being malignant, i.e., 30% of all the cancers revealed in our study (eight pulmonary cancers, two lymphomas, one neuroendocrine tumor, one breast cancer and four other metastatic cancers for which the pulmonary CT scan identified secondary lesions: one prostatic cancer, one pancreatic cancer, one colon cancer and finally one metastatic cancer with unknown primitive tumor).

In our cohort, 700 *abdominal ultrasounds* (US) were performed, of which 14 found suspicious abnormalities for a total of six eventually diagnosed cancers, i.e., 0.86% of all examinations carried out (three bladder cancers, one ovarian cancer, one renal cancer and one pancreatic cancer). Furthermore, 171 *thorax, abdomen and pelvis CT scans* (TAP CT) were performed; 23 showed suspicious abnormalities. Among them, 10 patients (i.e., 5.85% of all TAP CT scans performed) were diagnosed with cancer (two pancreatic cancers, two hematological malignancies, one lung cancer, one ovarian cancer, one bladder cancer, one hepatic cancer, one neuroendocrine cancer and one carcinoma of unknown primary origin), of which seven were metastatic at diagnosis.

Of the 492 patients who had a *fecal occult blood test* (FOBT), 138 had a positive result, of whom two patients (i.e., 0.41% of all tests performed) were diagnosed with colorectal cancer (one metastatic).

A total of 591 *serum protein electrophoresis* (SPEP) procedures were carried out, 27 of which showed a monoclonal spike. A malignant hematologic disease was diagnosed in four patients (i.e., 0.7% of SPEP performed), including two multiple myelomas, one myeloproliferative neoplasm and one Waldenström macroglobulinemia.

In our study, 372 *PSA* assays were performed, and 50 patients had a positive PSA test (beyond the threshold of 4 ng/mL), for a total of five confirmed cancers, i.e., 10% of all positive tests and 1.34% of the assays performed (three were already metastatic at diagnosis).

A total of 190 *mammograms* were performed, of which two showed pathological findings, i.e., 1.05% of all mammograms performed. The two patients who had an abnormal mammogram were diagnosed with breast cancer, of whom one had already metastasized.

### 3.3. Factors Associated with Occult Cancer

Factors associated with occult cancer at the moment of VTE diagnosis were identified through univariate analysis and integrated into a multivariate logistic regression model, resulting in two significant risk factors for occult cancer: age greater than 65 years old (OR 2.58, CI 95% 1.26–5.72; *p* = 0.0132) and the concomitant presence of lower-limb and unusual site venous thrombosis (OR 4.48, 95% CI 1.00–15.3; *p* = 0.0280). Of note, the provoked nature of the index event was not associated with a reduced risk of occult cancer (Table 4).

### 3.4. One-Year Outcomes

**Figure 2:**
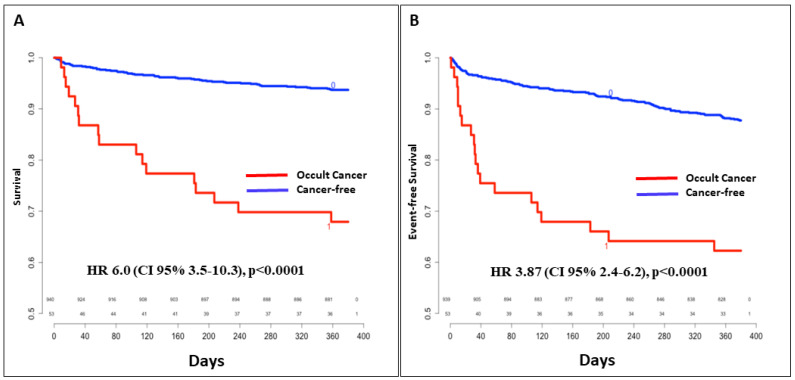
Overall survival (**A**) and event-free (VTE recurrence, major bleeding and all-cause death) survival (**B**) according to occult cancer status. CI: confidence interval; HR: hazard ratio; VTE: venous thromboembolism.

Overall, 136 patients had at least one complication from the composite criterion, including VTE recurrence, major bleeding and all-cause death, during the one-year follow-up (38% of the cancer patients versus 13.4% of cancer-free patients, HR 3.87 (CI95% 2.4–6.2, *p* < 0.0001). Eighty-three patients died during FU: 32% in the cancer group and 7% in the cancer-free population, HR 6.0 (CI95% 3.5–10.3, *p* < 0.0001) (Figure 2). Cancer evolution was the leading cause of death in the “occult cancer” group (10 out of 17 deaths).

## 4. Discussion

### 4.1. VTE-Associated Occult Cancer Incidence

VTE may be the first manifestation of cancer. In our study, the incidence of VTE-revealed occult cancers was low (5.3%) but similar to the incidences shown in recent studies [8,10,19]. In a population of more than 50,000 symptomatic acute VTE patients from the RIETE (Registro Informatizado Enfermedad TromboEmbólica) registry, 4% were diagnosed with cancer within one month of VTE, while, in a subgroup of 5863 patients with a 2 year-FU, 8% appeared to have occult cancer at the time of VTE diagnosis [20]. In a meta-analysis of recently published studies (2010–2016) of over 2000 patients with unprovoked VTE, Van Es et al. found an incidence of 5–6% of occult cancer after 1 year of FU, while previous studies have found a higher incidence (around 10%) [9,21].

### 4.2. Cancer Types Associated with VTE

Our study showed that the most frequently discovered cancers after acute VTE were gastrointestinal (23%), urinary (23%), pulmonary (19%) and hematological neoplasia (19%). Half (51%) were already metastasized at diagnosis.

In the literature, the types of cancer discovered after acute VTE are the most frequent ones and not necessarily the most thrombogenic: lung, colorectal and breast [3]. However, their distribution is slightly different than in the general population; indeed, lung, colorectal and breast cancers are the most frequent occult cancer types discovered after acute VTE, while breast and prostate are the most frequent cancers in the general population [3]. Thus, although highly thrombogenic cancers are more frequent in VTE patients than in the general population, they appear less incident than the most frequent cancers.

### 4.3. Accuracy of Cancer Screening Strategies in the Context of Acute VTE

When performing cancer screening following VTE, clinicians aim for earlier cancer detection and improved prognosis in terms of life expectancy. Screening strategies vary from one report to another. The large heterogeneity of solid and hematologic cancer distribution after acute VTE accounts for difficulties in targeting organ-specific cancers in current screening strategies. However, most studies have failed to show any benefit for overall morbidity and mortality despite a higher number of initially detected cancers, possibly at an earlier stage [11]. As such, current guidelines recommend limited assessment (medical history, physical examination, laboratory tests, chest X-ray) associated with age- and sex-specific procedures (mammography between 50 and 75 years old, cervical Pap test in women aged 21–65 years, FOBT in individuals aged 50–75 years, PSA or prostate examination in men aged 55 to 70 years and low-dose thoracic CT for smokers aged more than 55 years old).

In our cohort, the most efficient screening tests were the initial CTPA, involved in the diagnosis of 30% of all cancers, and the TAP CT (when performed), responsible for 19% of all cancer diagnoses. The high detection rate for cancers on initial CTPA makes it an interesting examination to prioritize over a pulmonary VQ scan for the diagnosis of PE. Most studies investigating limited versus extensive screening have included abdominal imaging and sometimes whole-body ^18^F-FDG PET/CT. The SOMIT randomized clinical trial has shown a relative risk of 9.7 (95%CI 1.3–36.8) of being diagnosed with cancer during a 2-year FU in the extensive versus limited screening group, but with no impact on survival. The extensive screening comprised abdominal ultrasound, abdominal and pelvic CT, gastroscopy, colonoscopy, sputum cytology, hemoccult, tumor markers, mammography and Pap smear for women [6]. Conversely, the SOME randomized clinical trial, in a larger population (854 patients), failed to prove any difference between limited (chest X-ray, mammography, Pap testing, PSA) and extensive strategies (limited + abdominal and pelvic CT with virtual colonoscopy and gastroscopy) in terms of early cancer detection or cancer-related mortality [10]. Moreover, Robin et al. showed in a meta-analysis that extensive screening (using CT and/or PET/CT) versus standard screening did not reduce the overall mortality after a VTE episode [22]. More recent studies, including ^18^F-FDG PET/CT as a screening tool, detected more cancers but were not conclusive because of an insufficient number of patients included (MTEVP study) [8]. PET/CT has shown, in a recent individual patient data meta-analysis, a high negative predictive value (98.9% (95%CI 94.3–99.7) but quite low positive predictive value (17.9%, 95%CI 8.5–33.6), which led to inconclusive results [23].

In an individual participant data meta-analysis (IPDMA), including 10 studies enrolling patients after the year 2000, Van Es et al. showed a twofold higher (OR 2.0, CI95% 1.2–3.4) probability of malignancy detection with extended strategies at the initial period, but the difference became insignificant after one-year FU (OR 1.4, CI95% 0.89–2.1) [21].

Moreover, *abdominopelvic US* showed poor diagnostic performance in our study and should probably not be carried out systematically.

Furthermore, in this VTE population, FOBT allowed the diagnosis of two colorectal cancers, i.e., 1.4% of the positive tests, which is a lower proportion as compared to mass screening policies in the French population, where the positive predictive value amounted to 4.7%. The large number of false positives could be explained by the anticoagulant treatment, triggering gastro-intestinal occult bleeding. These bleedings most frequently occur on colic polyps of low- or high-grade dysplasia, whose neoplastic conversion potential remains to be assessed. However, anticoagulant treatment may be the triggering factor leading to neoplasia discovery, as shown by the COMPASS trial, in which gastro-intestinal bleeding with antithrombotic drugs was associated with higher rates of new cancer diagnosis [24].

Mass screening with mammograms is the most effective method to detect breast cancer in the general population [25]. In our study, the cancer detection rate for mammograms was 1%, which is a higher than the detection rate of the mass screening carried out in the Alsatian population in 2011, where the detection rate ranged from 0.47 to 0.62% [26]. Recently, the American Cancer Society recommended continuing screening in healthy women at average risk of breast cancer with no age limit [27]. Faced with a detection rate almost twice as high as that carried out within the framework of mass screening, this examination may be of interest in the etiological assessment of VTE in the population falling within the mass screening age target (50–74 years old) and probably beyond the age limit of 74.

### 4.4. Risk Factors Associated with Occult Cancer Detection

In our cohort, two conditions were associated with higher risk of occult cancer: age over 65 years old and the association of limb DVT with unusual site thrombosis. Age represents the most recognized risk prognosticator of occult cancer (6.7% of cancer in VTE patients aged more than 50 years old versus 1% in patients younger than 50 years) [28]. Certain DVT characteristics, such as simultaneous multiple thrombus locations and unusual site thrombosis, have also commonly been associated with a higher risk of cancer, and this association was also found in our population [29]. In contrast, we did not find any association between the severity of PE and the presence of an underlying neoplasia.

Moreover, in this analysis of the REMOTEV registry population, we chose to not exclude provoked VTE events. Indeed, cancer risk after unprovoked VTE is generally considered to be higher than after a thromboembolic provoked event (7.6% versus 1.9%) [9]. However, several studies have shown no difference between cancer incidence after provoked and unprovoked VTE: one third of occult cancers in RIETE were found in patients with provoking risk factors. In a subgroup analysis of the RIETE registry, Rossel et al. found a high cumulative incidence of cancer in both unprovoked and provoked VTE cases (8.5% and 4.8%, *p* = 0.12) [30]. Furthermore, a similar effect was observed in the Hokusai VTE trial population, with similar cancer risks between provoked and unprovoked events (2.1% vs. 1.8%) [31].

Furthermore, we did not observe an association between active smoking and the discovery of cancer, which could be explained by the low number of active smokers (two patients) in the cancer group.

Several studies have explored the specific situations that could necessitate an extensive screening strategy. According to Robin et al., age over 50 years old, male sex, leukocytosis and thrombocytosis are trigger factors for extensive screening [8]. In a pos-hoc analysis of the SOME trail, Ihaddadehne et al. found a relevant correlation between age > 60 years old (HR 3.11; 1.41–6.89), VTE history (HR 3.20; 1.19–8.62) and tobacco consumption (HR 2.80; 1.24–6.33) and occult cancer [28]. In the RIETE registry, age > 70 years old, male sex, chronic heart disease, anemia and thrombocytosis were correlated to occult cancer, while VTE history and post-surgery status were “protective” factors [20,32].

Two predictive scores have been developed: the RIETE and SOME risk models. Higher age is the only common factor between these two scores. However, the discriminant capacity of these scores is low and their use in clinical practice to guide screening strategies is not recommended [33].

### 4.5. One-Year Prognosis

In our study, patients with VTE and newly diagnosed cancer had sixfold increased mortality during the first year following VTE. Similar rates of all-cause death were observed in a population-based study from Denmark, reporting a fivefold reduction in survival in the presence of concomitant VTE and cancer as compared to VTE alone [34]. As such, VTE appears as a strong prognosticator at 1 year for all cancer types, but the correlation between thrombus load and survival remains poorly studied [35].

### 4.6. Strengths and Limitations

Some limitations of our analysis need to be acknowledged. First, our study had a monocentric, observational design. Second, cancer detection was retrospectively assessed. Third, performance evaluation for each screening test should be interpreted with precaution since all patients did not have all tests, and this screening pattern only reflects current practices. Strengths of this study include the large number of unselected hospitalized VTE patients, the exclusion of all patients with a history of cancer—who could have biased the analysis as they benefit from regular cancer follow-up examinations—and the comprehensive collection of baseline characteristics. Moreover, despite a low number of detected cancers, our study brought some interesting information concerning the poor diagnostic value of certain explorations, such as abdomino-pelvic ultrasound and thoracic X-ray. Thus, a cost-effectiveness analysis for each individual examination, in the clinical VTE context, would be interesting.

## 5. Conclusions

In REMOTEV, VTE-revealed occult cancer prevalence was low but similar to recent reports and associated with higher age, multiple thrombotic sites and worse prognosis. Initial CTPA and mass screening approaches (FOBT, mammograms, PSA) seem to be efficient in detecting cancer after acute VTE, even beyond the fixed age limit, but screening must apply to a selected population, particularly in patients over 65 years, although their net clinical benefit is still under debate. Further studies assessing the survival benefit of neoplasia screening in elderly patients should be performed.

## Figures and Tables

**Table 1:** Baseline characteristics according to occult cancer status.

	Total N = 993 M(IQR)/N(%)	Occult Cancer N = 53 M(IQR)/N(%)	Absence of Cancer N = 940 M(IQR)/N(%)	*p*
**Demographic data**				
Age Age > 65 years	67 (50–78) 519 (52.3)	73 (66–82) 41 (77.4)	66 (49–78) 478 (50.9)	*0.00119* *0.0003*
Female	528 (53.2)	27 (50.9)	501 (53.3)	0.84
BMI (N = 911)	27 (24–31)	27 (24.5–30)	27 (23–31)	0.66
**Cardiovascular risk factors**				
Arterial hypertension	486 (48.9)	27 (50.9)	459 (48.8)	0.87
History of smoking (active or former) (N = 965) Active	403 (41.8) 158 (16.4)	21 (40.4) 2 (3.8)	382 (41.8) 156 (17.1)	0.95 *0.024*
Diabetes	146 (14.7)	6 (11.3)	140 (14.9)	0.60
Dyslipidemia	298 (30)	16 (30.2)	282 (30)	1
Obesity (N = 911)	313 (33.3)	15 (29.4)	298 (33.5)	0.65
Héredity	45 (4.5)	1 (1.9)	44 (4.7)	0.50
**Comorbidities**				
Ischemic heart disease	44 (4.4)	2 (3.8)	42 (4.5)	1
Stroke/transient ischemic attack	61 (6.1)	4 (7.5)	57 (6.1)	0.56
Lower-limb arteriopathy	29 (2.9)	2 (3.8)	27 (2.9)	0.66
Chronic obstructive pulmonary disease	52 (5.2)	3 (5.7)	49 (5.2)	0.75
Atrial fibrillation	59 (5.9)	3 (5.7)	56 (5.9)	1
Chronic kidney disease	513 (51.7)	38 (71.7)	475 (50.5)	*0.003*
60 ≤ eGFR MDRD < 90 mL/min/1.73 m^2^	347 (34.9)	27 (50.9)	320 (34)	*0.018*
30 ≤ eGFR MDRD < 60 mL/min/1.73 m^2^	138 (13.9)	10 (18.9)	128 (13.6)	0.38
15 ≤ eGFR MDRD < 30 mL/min/1.73 m^2^	23 (2.3)	1 (1.9)	22 (2.3)	1
eGFR MDRD < 15 mL/min/1.73 m^2^	5 (0.5)	0	5 (5.3)	1
Cognitive disorders	105 (10.6)	3 (5.7)	102 (10.9)	0.35
Psychiatric disorders	132 (13.3)	5 (9.4)	127 (13.5)	0.53
**VTE risk factors**				
Fracture/orthopedic surgery	35 (3.5)	1 (1.9)	34 (3.6)	1
Surgery with anesthesia > 30 min	78 (7.9)	1 (1.9)	78 (8.2)	0.11
VTE history	319 (32.1)	20 (37.7)	299 (31.8)	0.45
Recent hospitalization for heart/respiratory failure < 3 months				
Myocardial infarction < 3 months	12 (1.2)	1 (1.9)	11 (1.2)	0.48
Spinal cord injury	7 (0.7)	0	7 (7.4)	1
Thrombophilia	1 (1)	0	1 (1)	1
Paralysis	49 (4.9)	1 (1.9)	48 (5.1)	0.51
Central catheter	6 (0.6)	0	6 (0.6)	1
Oral contraception/hormone replacement therapy/in vitro fertilization	2 (0.2)	0	2 (0.2)	1
Pregnancy				
**Antithrombotic treatment at admission**				
Antiplatelet	179 (18)	10 (18.9)	169 (17.9)	1
Anticoagulant	64 (6.4)	7 (13.2)	57 (6.1)	0.07

BMI: body mass index; eGFR: estimated glomerular filtration rate; IQR: interquartile range; M: median; MDRD: modification of diet in renal disease; N: number; VTE: venous thromboembolism.

**Table 2:** Characteristics of the VTE index event.

	Total N = 993 M(IQR)/N(%)	Occult Cancer N = 53 M(IQR)/N(%)	Absence of Cancer N = 940 M(IQR)/N(%)	*p*
**Index event**				
PE	868 (87.4)	46 (86.8)	822 (87.4)	1
PE only	276 (27.8)	10 (18.9)	266 (28.3)	0.18
PE + DVT	592 (59.6)	36 (67.9)	556 (59.1)	0.26
DVT only	125 (12.6)	7 (13.2)	118 (12.6)	1
D-dimer (N = 764)	3280 (1800–7570)	3200 (2085–7770)	3280 (1800–7562)	0.99
Provoked	340 (35.1)	11 (33.3)	329 (35.2)	0.97
Length of hospital stay	6 (4–8)	8 (6–13)	6 (4–8)	*<0.0001*
**DVT extension**				
DVT lower limbs	683 (68.8)	42 (79.2)	641 (68.2)	0.12
Distal	198 (19.9)	15 (28.3)	183 (19.5)	0.16
Proximal	380 (38.3)	19 (35.8)	361 (38.4)	0.82
Bilateral	105 (10.6)	8 (15.1)	97 (10.3)	0.46
USVT	56 (5.6)	4 (7.5)	52 (5.5)	0.53
Concomitant DVT lower limbs and USVT	22 (2.2)	3 (5.7)	19 (2)	0.08

DVT: deep vein thrombosis; IQR: interquartile range; M: median; N: number; PE: pulmonary embolism; USVT: unusual site venous thrombosis.

**Table 3:** Diagnostic performance of cancer detection investigations.

Type of Exam	Total	Negative Exam	Positive Exam	Cancer Diagnosed	Cancer Diagnosed/Positive Exam	Cancer Diagnosed/Total
**Limited screening examinations**						
CTPA	627	NA	NA	16	NA	2.55%
Chest X-ray	726	725	1	0	0	0
FOBT	492	354	138	2	1.4%	0.41%
PSA	372	322	50	5	10%	1.34%
Mammograms	190	188	2	2	100%	1.05%
**Extended screening examinations**						
Abdomino-pelvic US	700	686	14	6	42.8%	0.86%
TAP CT	171	148	23	10	43.5%	5.85%
SPEP	591	564	27	4	14.8%	0.67%

CTPA: computed tomography pulmonary angiogram; FOBT: fecal occult blood test; NA: not available; PSA: prostatic-specific antigen; SPEP: serum protein electrophoresis; TAP CT: thorax abdomen and pelvis computed tomography; US: ultrasound.

**Table 4:** Univariate and multivariate analysis of factors associated with the discovery of cancer during an episode of VTE.

Variable	Univariate Analysis			Multivarate Analysis		
	OR	95%CI	*p*	OR	95%CI	*p*
Age > 65 years	3.47	1.83–7.17	0.0003	2.58	1.26–5.72	*0.0132*
Active smoking	0.19	0.03–0.63	0.024	0.33	0.05–1.19	0.150
Chronic kidney disease	2.48	1.37–4.70	0.003	1.59	0.83–3.18	0.17
Anticoagulation	2.11	0.61–5.55	0.07	2.09	0.58–5.81	0.19
Concomitant DVT of the lower limbs and USVT	2.91	0.66–8.89	0.08	4.48	1.00–15.3	*0.0280*

CI: confidence interval; DVT: deep vein thrombosis; OR: odds ratio; USVT: unusual site venous thrombosis.

## Data Availability

Not applicable.
